# The Treatment of Fistulous Withers

**Published:** 1901-08

**Authors:** W. H. Wheeler

**Affiliations:** Stamford, N. Y.


### REPORTS OF CASES.

### THE TREATMENT OF FISTULOUS WITHERS.

By W. H. Wheeler, V.S.,
STAMFORD, N. Y.

It is not my intention in writing this article to offer anything new in the treatment of fistula, but simply to give my experience with a remedy which I have found in my practice to give good results in these most troublesome cases, and I think I am safe in speaking of them as troublesome, as that seems to be the opinion of all veterinarians with whom I have talked on the subject. They as well as myself have tried every treatment that they could think of or read about, and all joined in saying that fistula is a most stubborn affection to manage.

Protargol is the remedy which I have found to give the most satisfactory results, and I consider it a specific in these cases. It is a derivative of silver, a yellowish light powder, and freely soluble in water. It is not irritating like the nitrate or other caustics, and it is in this respect that I regard it superior to any other known remedy. My first experience with this drug was during last summer. I had been treating a two-year-old stallion colt for fistulous withers for about six weeks, and had used the knife freely, with setons, caustics, etc., but the improvement was very slow. Having heard of the value of protargol in this condition, and at the time having an ounce on hand, I concluded to give it a trial. So to this ounce of protargol I added five ounces of glycerin and four ounces of water, and directed the owner to inject a small quantity of this fluid, after cleansing thoroughly, twice per day. About twelve days after this I was driving by the owner's place, so thought I would stop for a few minutes to see my patient and how the new remedy was working. The colt was brought out on the floor, and to my surprise the swelling was all gone. I saw that all it then needed was to remove the seton and use some mild antiseptic, and under the use of a weak carbolic solution applied twice per day for about two weeks the fistula entirely healed, and has remained sound and smooth ever since. After seeing how nicely the drug worked in this case, I concluded it would be a good article to have on hand, and ordered a supply.

My next case of this kind occurred on July 25th, when I was called to see a sorrel mare, seven years old, with very bad fistulous withers; both sides and the top of the withers were swollen very badly, and on one side was a running sore with a very offensive odor. On asking the farmer how long the case had been in this condition, he informed me that for about three weeks he had noticed the swelling, and had opened one side with his jack-knife about a week previously. Casting the animal and making a thorough examination of the parts, I saw at once that I had a very bad case to deal with. Pus had buried beneath the scapula on one side; the ligamentum nuchæ and all the muscles of the parts were involved, and were simply one mass of pipes. I laid the fistula well open with a knife, and inserted a seton in each side and one under the ligament, which gave it a good dependent opening. I ordered protargol solution to be used twice per day after cleansing well with carbolic-acid water. I did not see the case again until August 18th, when I removed the seton, and kept up the same treatment until September 18th, when the mare was entirely cured and commenced to work. She is well and sound at the present time.

About August 1st one of my neighbors came to my office stating that his family horse had an enlargement or swelling on the top of his head, which had been there for a few days, and did not seem to come down. I went over to his barn and examined his horse, and told him that the animal had a poll-evil, which seemed to daze him at first, as if I had told him he had been exposed to smallpox and was coming down with the disease. He thought from what he had heard of poll-evil that it would be cheaper for him to kill the horse than to have it treated. After telling him that this trouble was unpleasant to treat, and sometimes very tedious, but that I could probably cure it, he concluded to have the case treated, and not to destroy the horse for a while. He had been using hot cloths on the enlargement, and it was ready to open. After incision I placed a seton on each side and one over the top. I then crowded in a few plugs of nitrate of silver, corrosive sublimate, and protargol, which gave me an abundant discharge of pus in about twelve hours. The next day I instructed him to syringe it out well twice daily with carbolic-acid water, after which to apply some of the protargol solution. This treatment was continued until September 12th, when the horse was entirely well, and is still so to-day.

I have now a case of fistulous withers which has been treated with the ordinary remedies. The case was brought to me five days ago. The affected part is a solid mass of pipes and badly swollen. I have used the knife quite freely, and I am now employing protargol solution, and expect to send the horse away in six weeks' time.

To those who are not familiar with protargol I would advise them to try it on their fistulous cases, and I am sure that after once using this remedy nothing else will take its place. It seems to be a specific for the treatment of fistulous tracts, not only healing them quickly, but permanently.

